# Plant Secondary Metabolites Modulate Insect Behavior-Steps Toward Addiction?

**DOI:** 10.3389/fphys.2018.00364

**Published:** 2018-04-11

**Authors:** Michael Wink

**Affiliations:** Institute of Pharmacy and Molecular Biotechnology, Heidelberg University, Heidelberg, Germany

**Keywords:** plant secondary metabolites, pharmacology, toxicology, plant-insect interactions, neurotoxicity, psychoactive natural products

## Abstract

Plants produce a diversity of secondary metabolites (PSMs) that serve as defense compounds against herbivores and microorganisms. In addition, some PSMs attract animals for pollination and seed dispersal. In case of pollinating insects, PSMs with colors or terpenoids with fragrant odors attract pollinators in the first place, but when they arrive at a flower, they are rewarded with nectar, so that the pollinators do not feed on flowers. In order to be effective as defense chemicals, PSMs evolved as bioactive substances, that can interfere with a large number of molecular targets in cells, tissues and organs of animals or of microbes. The known functions of PSMs are summarized in this review. A number of PSMs evolved as agonists or antagonists of neuronal signal transduction. Many of these PSMs are alkaloids. Several of them share structural similarities to neurotransmitters. Evidence for neuroactive and psychoactive PSMs in animals will be reviewed. Some of the neuroactive PSMs can cause addiction in humans and other vertrebrates. Why should a defense compound be addictive and thus attract more herbivores? Some insects are food specialists that can feed on plants that are normally toxic to other herbivores. These specialists can tolerate the toxins and many are stored in the insect body as acquired defense chemicals against predators. A special case are pyrrolizidine alkaloids (PAs) that are neurotoxic and mutagenic in vertebrates. PAs are actively sequestered by moths of the family Arctiidae and a few other groups of arthropods. In arctiids, PAs are not only used for defense, but also serve as morphogens for the induction of male coremata and as precursors for male pheromones. Caterpillars even feed on filter paper impregnated with pure PAs (that modulate serotonin receptors in vertebrates and maybe even in insects) and thus show of behavior with has similarities to addiction in vertebrates. Not only PA specialists, but also many monophagous herbivores select their host plants according to chemical cues i.e., PSMs) and crave for plants with a particular PSMs, again a similarity to addiction in vertebrates.

## Evolution and function of plant secondary metabolites^1^

Since the early days of plant evolution in the Devonian period, plants had to cope with herbivores, but also with bacteria, fungi and viruses around them. Plants cannot run away when attacked by an herbivore, nor do they possess an adaptive immune system as present in vertebrates against microbial infections (Wink, 1988, 2003).

Similar to the situation of other immobile or slow-moving organisms (amphibians, slugs, cnidarians, and sponges) plants invested into the production of a wide diversity of organic compounds, the so-called secondary metabolites (PSMs). The structures of PSMs underwent several rounds of selection; thus their structures were shaped in such a way that they could interfere with the metabolism, neuronal transmission or reproduction of an herbivore or microbe. In consequence, nearly all PSMs exhibit some sort of biological activity and PSMs support plants to ward off herbivores and microbial infections (Wink, 1988, 2003). Plants also employ other strategies in this context, such as an impenetrable bark and cuticles, thorns, spikes and stinging hairs; furthermore, plants possess the capacity of open growth. Thus, they can renew parts that had been damaged by an herbivore.

Plants produce a substantial structural diversity of PSMs, such as alkaloids, amines, cyanogenic glucosides, glucosinolates, non-protein amino acids, organic acids, terpenoids, phenolics, quinones, polyacetylenes, and peptides. Over 100,000 individual structures have been elucidated already (Wink, 1988, 2003). Plants do not produce a single compound for defense, but usually a complex mixture of PSMs from different structural classes that can attack multiple molecular targets at the same time and often in a synergistic fashion (Wink, 2008, 2015; Mason and Singer, 2015). The composition of these mixtures is not fixed, but varies in terms of both concentration and composition. Thus, mixtures differ between organs, developmental stages and within populations. We had previously suggested that this variation is an important strategy to avoid the adaptation and resistance of herbivores and pathogens against the chemical defense. It is widely known from medicine, that treatment of bacteria or viruses with a single drug will give raise to resistant strains in a rather short time (e.g., antibiotic resistance).

PSMs evolved as an important line of defense, but some of them are further used for other purposes. Flowering plants often employ insects as pollinators, and also a few other arthropods and vertebrates. These pollinators are attracted to flowers by their color or smell; color is usually due to the production of flavonoids, anthocyanins, or carotenoids, whereas terpenoids, amines and phenylpropanoids exhibit distinctive odors that are recognized by pollinators (not necessarily by all animals). However, pollinators should be attracted to flowers but should not eat them. Thus, the attractant PSMs and other compounds are toxic and deterrent for a pollinator that tries to feed on flowers. Instead, flowers produce sugar-rich nectar as a reward for pollinating animals that they normally prefer over other flowering material (Wink, 1988, 2003; Detzel and Wink, 1993). Plants try to disperse their seeds beyond the direct neighborhood of the producing mother plants. Also in this context, animals are being manipulated as fruit–and seed dispersers. Mature fruits are usually sweet and show attractive colouration and smell. Fruit-eating animals (frugivores) are adapted to eat ripe fruits; but they do not destroy the seeds, that pass the intestinal tract without harm. Furthermore, as frugivores will deposit their faces far away from the fruiting tree, the seeds become dispersed and furthermore they are dropped together with potential fertilizers. Some PSMs also serve the producing plants directly as antioxidants, nitrogen storage compounds or for UV protection. Thus, most PSMs have multiple functions for a plant producing them (Wink, 1988, 2003).

A special case is the production of PSMs that interfere with the nervous system in animals. In vertebrates, several small-molecule neurotransmitters are known that modulate the activity of neuroreceptors (Wink, 2000). Among the most important neurotransmitters are acetylcholine, GABA, serotonin, dopamine, adrenaline, noradrenaline, adenosine, histamine, glutamate, and endorphins. Some of the PSMs that mimic the structure of neurotransmitters are CNS stimulants, others psychedelic and hallucinogenic (especially those binding to serotonin and dopamine receptors). Because herbivores that feed on psychoactive PSMs, often become addicted to the drugs, such compounds appear to be counterproductive, as they will attract herbivores. However, in the wild, the survival of an intoxicated herbivore is probably quite short. It will either fall from trees and rocks or will be an easy prey for the predators which are abundant in most ecosystems.

## Pharmacology and toxicology of plant secondary metabolites

Among alkaloids, several modulate neuronal signal transduction and are thus often toxic for herbivores. Ion channels, neurotransmitter receptors, neurotransmitter inactivating enzymes and transporters play an important role. Examples for alkaloids, known to interfere with these targets (mostly in vertebrates) are documented in Table 1.

**Table 1:** Examples for alkaloids and other PSMs that modulate neuronal signal transduction (more details in Wink, 2000; Wink and Schimmer, 2010).

**Target**	**Alkaloids**	**Activity**
**ION CHANNELS**		
	Aconitine	Agonist
	Ajmaline	Antagonist
	Berbamine	Antagonist
	Capsaicin	antagonist
	Cocaine	Antagonist
	Dicentrine	Antagonist
	Ervatamine	Antagonist
	Glaucine	Antagonist
	Hirsutine	Antagonist
	Liriodenine	Antagonist
	Paspaline	Antagonist
	Phalloidin	antagonist
	Quinidine	Antagonist
	Ryanodine	Agonist
	Sparteine	Antagonist
	Strychnine	Antagonist
	Veratrine	Agonist
	Vincamine	Antagonist
	Zygademine	Agonist
**NEUROTRANSMITTER RECEPTORS**		
**Nicotinic acetylcholine receptor (nAChR)**		
	Anabasine	Agonist
	Boldine	Antagonist
	Coniine	Agonist
	Cytisine, lupanine	Agonist
	Erythrina alkaloids	Antagonist
	Methyllycaconitine	Antagonist
	Nicotine	Agonist
	Tubocurarine	Antagonist
**Muscarinic acetylcholine receptor (mAChR) (GPCR)**		
	Arecoline	Agonist
	Cryptolepine	Agonist
	Ebeinone	Antagonist
	Himbacine	Antagonist
	Hyoscyamine, scopolamine	Antagonist
	Muscarine	Agonist
	Pilocarpine	Agonist
	Sparteine	agonist
**Dopamine receptor (GPCR)**		
	Agroclavine	Agonist
	Bulbocapnine	Antagonist
	Epinine	Agonist
	Ergot alkaloids	Agonist
	Loline	Agonist
	Salsolinol	Agonist
	Tyramine	Antagonist
**Serotonin receptor (mostly GPCR)**		
	Akuammine	Antagonist
	Bufotenine	Agonist
	Confusameline	antagonist
	Ergot alkaloids	Antagonist
	Harman and other harmine alkaloids	Agonist
	Liridinine	Antagonist
	Mescaline	Agonist
	Mitragynine	Agonist
	N-Methyltryptamine	Agonist
	Psilocine	Agonist
**Adenosine receptor (GPCR)**		
	Caffeine, theobromine, theophylline	Antagonist
**GABA receptor (mostly ICR)**		
	Bicuculline	Antagonist
	Corlumine	Antagonist
	Hydrastine	Antagonist
	Muscimol	Agonist
	Securinine	Antagonist
**Glutamate receptor (mostly ICR)**		
	Acromelic acid	Agonist
	Domoic acid	Agonist
	Ibogaine	Antagonist
	Ibotenic acid	Agonist
	Kainic acid	Agonist
	Nuciferine	Antagonist
	Quisqualic acid	Agonist
	Willardiine	Agonist
**Noradrenaline receptor (GPCR)**		
	Ajmalicine	Antagonist
	Berbamine	Antagonist
	Berberine	Antagonist
	Boldine	Antagonist
	Bulbocapnine	Antagonist
	Corynanthine	Antagonist
	Crebanine	Antagonist
	Dispegatrine	Antagonist
	Ergot alkaloids	Agonist / antagonists
	Glaucine	Antagonist
	Octopamine	Agonist
	Predicentrine	Antagonist
	Yohimbine	Antagonist
**Endorphine receptor (GPCR)**		
	Akuammine	Agonist
	Ibogaine	Agonist
	Mitragynine	Agonist
	Morphine	Agonist
**INACTIVATION OF NEUROTRANSMITTERS**		
**Acetylcholine esterase**		
	Berberine	Antagonist
	Galantamine	Antagonist
	Harmaline	Antagonist
	Huperzine	Antagonist
	Physostigmine	Antagonist
	Sanguinarine	Antagonist
	Solanum alkaloids	Antagonist
	Vasicinol	Antagonist
**Monoamine oxidase (MAO)**		
	Alstovenine	Antagonist
	Carnegine	Antagonist
	*N,N-*dimethyltryptamine	Antagonist
	Harmaline and other harman alkaloids	Antagonist
	Saracodine	Antagonist
	Salsolidine	Antagonist
**TRANSPORTER FOR NEUROTRANSMITTERS**		
	Annonaine	Inhibition of DA reuptake
	Arecaidine	Inhibition of GABA reuptake
	Cocaine	Inhibition of DA uptake
	Ephedrine	Release of NA from synaptic vesicles; inhibition of NA reuptake
	Ibogaine	Modulates DA, NA and 5-HT transporters in synaptic vesicles
	Norharman	Inhibitor of DA and tryptamine uptake
	Reserpine	Depletes stores of NA and 5-HT in synaptic vesicles
	Salsolinol	Inhibitor for uptake of biogenic amine neurotransmitters
	Veratramine	Releaser and uptake inhibitor for 5-HT
**ION PUMPS**		
**Na**^+^**/K**^+^**-ATPase**		
	Ouabain and other cardiac glycosides	Inhibitor

In case of neurotransmitter receptors, some are ion channel coupled receptors (nAChR, 5-HT3, NMDA, AMPA, kainate, GABAA) (= ICR), the other metabotropic receptors coupled with G-protein (GPCR)

When PSMs modulate elements of neuronal signal transduction, the concentrations of neurotransmitters, the activity of neurotransmitter receptors or their expression can be changed. This can lead to severe changes in physiology and often in the behavior of an animal. Addiction can be one of them.

Many PSMs can modulate the bioactivity and/or 3D structure of proteins. Among them are some specific inhibitors (such as colchicine, inhibiting microtubule assembly). The majority of the widely distributed phenolic compounds can modulate the 3D structure of proteins by forming multiple hydrogen and ionic bonds with them (Table 2; Wink, 2008, 2015). In addition, some PSMs possess chemically reactive functional groups by which they can form covalent bonds with amino, sulfhydryl or hydroxyl groups of amino acid residues of proteins (Table 2). Lipophilic terpenes can assemble in the inner hydrophobic core of globular proteins that thus can change their 3D structures.

**Table 2:** PSMs interfering with proteins (more details in Wink, 2008, 2015; Wink and Schimmer, 2010).

**Activity**	**PSMs**	**Examples**	**Comments**
**COVALENT BONDS WITH PROTEINS**			
With SH-groups	With SH groups	Allicin and similar PSMs	
With amino groups	With epoxy groups		
	With exocyclic methylene groups	Sesquiterpene lactones	
**Non-covalent bonding with proteins**	With amino groups	Phenolics, polyphenols	Hydrogen and ionic bonds
	With hydroxyl groups	Phenolics, polyphenols	Hydrogen and ionic bonds
	With lipophilic sites	Lipophilic terpenes	Hydrophobic attraction

A special case of protein inhibitors are those which can interfere with protein synthesis in ribosomes, such as lectins (e.g., ricine, abrine), emetine, and lycorine (Wink and Schimmer, 2010; Wink, 2015).

Biomembranes that surround all living cells and intracellular compartments, are the target for lipophilic PSMs (Table 3). They can be trapped inside the biomembrane and thus change its fluidity and permeability. Typical lipophilic PSMs include mono-, sesqui-, di-, and triterpenes, steroids, mustards oils, and phenylpropanoids. A special case are saponins that consist of a lipophilic steroid or triterpene moiety and a hydrophilic sugar chain. These compounds are amphiphilic and can lyse biomembrane by complexing membrane cholesterol (Table 3). Also antimicrobial peptides (AMPs) that are part of the innate immune system of most organisms, interfere with biomembranes of microbes but also of eukaryotic cells.

**Table 3:** PSMs interfering with biomembranes (more details in Wink, 2008, 2015; Wink and Schimmer, 2010).

**Activity**	**PSMs**	**Examples**	**Comments**
Increasing membrane fluidity	Lipophilic terpenoids	Monoterpene, sesquiterpenes	Membranes become leaky or disintegrate
Lysis of membranes	Triterpene and steroidal saponins		Saponins bind to membrane cholesterol and induce cell lysis
	AMPs	Widely distributed	Part of the innate immune system

Several PSMs can interfere with nucleic acid and enzymes that metabolize them (Wink and Schimmer, 2010). We can distinguish between DNA intercalating and DNA alkylating compounds (Table 4). Lipophilic, aromatic and planar PSMs (such as furanocoumarins, berberine, sanguinarine) can intercalate between the stacks of DNA-base pairs. Intercalators stabilize DNA and can prevent the activity of helicases and RNA polymerases; they can be mutagenic (because of frame shift), genotoxic, and cytotoxic (Table 4). Alkylating agents directly bind to nucleotide bases and form covalent bonds. They also lead to mutations and genotoxicity (Table 4).

**Table 4:** Examples for PSMs interfering with nucleic acids (DNA, RNA) (more details in Wink, 2008, 2015; Wink and Schimmer, 2010).

**Target**	**PSMs**	**Activity**	**Comments**
**DNA INTERCALATION**			
	Berbamine	Strong intercalator	Also inhibition of replication and ribosomal protein biosynthesis
	Berberine	Strong intercalator	Also inhibition of replication and ribosomal protein biosynthesis
	Dictamnine and other furaquinoline alkaloids	Strong intercalator	After UV activation also DNA alkylation
	Ellipticine	Strong intercalator	
	Harmine and other Harman alkaloids	Strong intercalator	Also inhibition of replication and ribosomal protein biosynthesis
	Psoralen and other furanocoumarins	Strong intercalator	After UV activation also DNA alkylation
	Rutacridone and other acridone alkaloids	Strong intercalator	After UV activation also DNA alkylation
	Sanguinarine	Strong intercalator	Also inhibition of replication and ribosomal protein biosynthesis
**DNA ALKYLATION**			
	Aristolochic acid	Mutagenic after metabolic activation	
	Cycasin	Methylazooxymethanol is the active mutagen	
	Furanoquinoline alkaloids	Mutagenic after metabolic activation	
	Ptaquiloside	Active after removal of glucose from glycoside	
	Safrole and other phenylpropanoids	Mutagenic after metabolic activation	
	Senecionine and other PAs	Mutagenic after metabolic activation	

## Plant–insect interactions

Among all multicellular living organisms, plants and insects exhibit the largest diversity with more than 1 million described arthropod taxa (mostly insects) and more than 350,000 plant taxa. Amongst eukaryotes, the diversity of plants and metazoans pales in comparison to the diversity amongst fungal taxa (albeit non-described so far; Yahr et al., 2016). Although flowering plants (angiosperms) evolved already during the Cretaceous, an extensive radiation took place at the start of the Tertiary, 66 million years ago. Evidence suggests that parallel to the angiosperm radiation, a radiation of insects set in as well. If both radiations were true co-evolutionary processes is an open debate. Many insects are pollinators, others are herbivorous. Among the herbivores, we can distinguish polyphagous species that feed on many plant species, oligophagous species that love a selection of plants and monophagous species that are adapted to individual species or species groups which produce similar PSMs (Ali and Agrawal, 2012; Mason and Singer, 2015).

The herbivorous insects had and still have to cope with the PSMs in their food plants (Detzel and Wink, 1993; Linde and Wittstock, 2011). They have evolved several mechanisms to tolerate or detoxify PSMs. Mostly, the generalists have very active enzymes that either inactivate (via CYP) or quickly eliminate (via ABC transporter) toxic PSMs. Another strategy is to feed not only on one plant, but to sample from several species (with low PSMs content) thus diluting any toxic effect. Often herbivores have a fast digestion, by which they absorb nutrients quicker than any toxins that are quickly eliminated in the feces. For detoxification, some herbivores obtain help from symbiotic intestinal microorganisms that often can degrade or inactivate toxic material (Pennisi, 2017).

From a plant side of view, the specialists have won the evolutionary arms race. They can harm their host plants severely if their numbers are large. This can be seen in areas where *Senecio jacobaea* plants (producing PAs) are abundant. If the PA specialist moth *Tyria jacobaeae* occurs in the same area, a *Senecio* population may suffer seriously. But even under these conditions *Tyria* will not completely wipe out its host plants (Wink and Legal, 2001). A predator–prey equilibrium will emerge in the long run.

## Utilization of plant secondary metabolites by insects

Among monophagous insects, several specialists have been described that apparently love their toxic host plants. These specialists often not only tolerate the toxic PSMs of the host plant, but actively sequester them in their body (Wink, 1992, 1993; Brown and Trigo, 1995; Hartmann and Witte, 1995; Hartmann, 1999, 2004; Petschenka and Agrawal, 2016). Thus, these specialists can store substantial amounts of toxic PSMs and use them for their own defense against predators (Mason and Singer, 2015). Such specialist have been described for toxic cardiac glycosides, aristolochic acids, cyanogenic glucosides, iridoid glucosides and several toxic alkaloids (aconitine, pyrrolizidines, quinolizidines) (Wink, 1992, 1993; Sime et al., 2000; Dobler, 2001; Zagrobelny and Møller, 2011; Kelly and Bowers, 2016; Petschenka and Agrawal, 2016). These specialist often exhibit warning colors, i.e., they are aposematic; and thus advertise their potential toxicity to any predator.

In most instances, we do not know how these specialists circumvent the inherent toxicity of PSMs. For some insects that sequester cardiac glycosides, it could be shown, that the binding site of their molecular target, the Na^+^, K^+^-ATPase, has been changed through point mutations in such a way, that cardiac glycosides no longer bind to it. Thus, Monarch butterflies can tolerate high concentrations of cardiac glycosides that would kill any poly- or oligophagous species (Holzinger et al., 1992; Holzinger and Wink, 1996; Dobler et al., 2012; Aardema and Andolfatto, 2016). In most other cases, we do not have a clear evidence, how an insensitivity has been accomplished.

## Addiction of insects to plant chemistry?

As mentioned above, monophagous species [mostly butterflies and moths, aphids and other hemipterans) only feed on a single particular plant species that produces a certain kind of toxin, such as cardiac glycosides, iridoid glycosides, glucosinolates, cyanogenic glucosides, or alkaloids [pyrrolizidine (PA), quinolizidine alkaloids (QA)] (Boppré, 1984; Wink, 1992, 1993; Brown and Trigo, 1995; Hartmann and Witte, 1995; Hartmann, 1999, 2004; Klitzke and Trigo, 2000; Laurent et al., 2005; Hilker and Meiners, 2011; Macel, 2011; Trigo, 2011; Cogni et al., 2012). If related plants produce similar toxins, such as in *Brassica* species that all produce glucosinolates, then even a monophagous species may feed on more than a single host plant because they love these particular PSMs. But they will not live on plants with different kinds of PSMs.

Who decides on the choice of a food plant? In most instances, it will be the female with fertile eggs that will search for its specific food plants that it can identify because of their typical PSMs profile. In case of plants from the family Brassicaceae that all produce glucosinolates (which release the often odorant mustard oils), it has been shown that the mustard oils guide a butterfly to its appropriate host plant (Renwick and Lopez, 1999). Apparently, specific odorant receptors have evolved in such butterflies (like Garden Whites, Pieridae) that are activated if the odorant from cabbage plants pass along their antennae. In this instance, the plant odorant appears to work like the pheromones that are used by insects to attract potential mates. Food consumption by larvae of *Pieris rapae* that love food plant with glucosinolates, has been compared with addiction in vertebrates (Renwick and Lopez, 1999).

A similar situation has been described from arctiid moths with sequester PAs, such as *Utetheisa* and *Creatonotus*. We have studied PAs in *Creatonotus* for several years in collaboration with Dietrich Schneider, who had discovered the strange relationship between moths and PAs (Boppré, 1986; Wink and Schneider, 1988, 1990; von Nickisch-Rosenegk et al., 1990; von Nickisch-Rosenegk and Wink, 1993). The caterpillars can be reared on artificial diets without PAs. But the hairy caterpillars of *Creatonotus gangis* and *C. transiens* clearly prefer any plant that produces PAs. Plants with other toxic alkaloids are usually avoided. The larvae appear to be addicted to PAs, because they will even chew filter paper that was impregnated with pure PAs. Normally, they would never touch filter paper, even when hungry. This indicates that PAs induce a very strong feeding stimulus, similar to the situation of the behavior of humans toward addictive drugs.

Addiction in humans implies a craving for a certain chemical whose consumption would confer happiness, good feeling or hallucinations. Addiction will change the personality of the consumer as a strong urge appears once the level of the addictive chemical has dropped in the body.

PAs are actively absorbed by the larvae: PAs mostly occur as polar PA N-oxides which cannot pass biomembranes by simple diffusion. There is evidence that transporter proteins exist at the gut epithelium that can transport the polar alkaloids into the haemolymph (Wink and Schneider, 1988). An alternative mechanism was also found, in that PA become reduced to the more lipophilic free base in the gut which can pass the membranes by simple diffusion. Once the alkaloids have reached the haemolymph, they will be re-oxidized to PA N-oxides (Wang et al., 2012). The PAs do not stay in the haemolymph, but are sequestered into the integument of the larvae (Egelhaaf et al., 1990; von Nickisch-Rosenegk et al., 1990; Wink et al., 1990; von Nickisch-Rosenegk and Wink, 1993), where they serve as defense compounds against predators (Martins et al., 2015).

However, the situation becomes more complex if we look closer into male and female larvae after metamorphosis into adult insects: In female larvae, PAs will be sequestered to some degree in the integument, but a large part is transferred to the orange colored eggs that thus gain chemical protection (von Nickisch-Rosenegk et al., 1990). PAs as a nuptial gift for the defense of the eggs has also been described for other arctiids *Utetheisa ornatrix* and *Cosmosoma myrodora* (González et al., 1999; Conner et al., 2000; Bezzerides and Eisner, 2002; Cogni et al., 2012).

**Figure 1:**
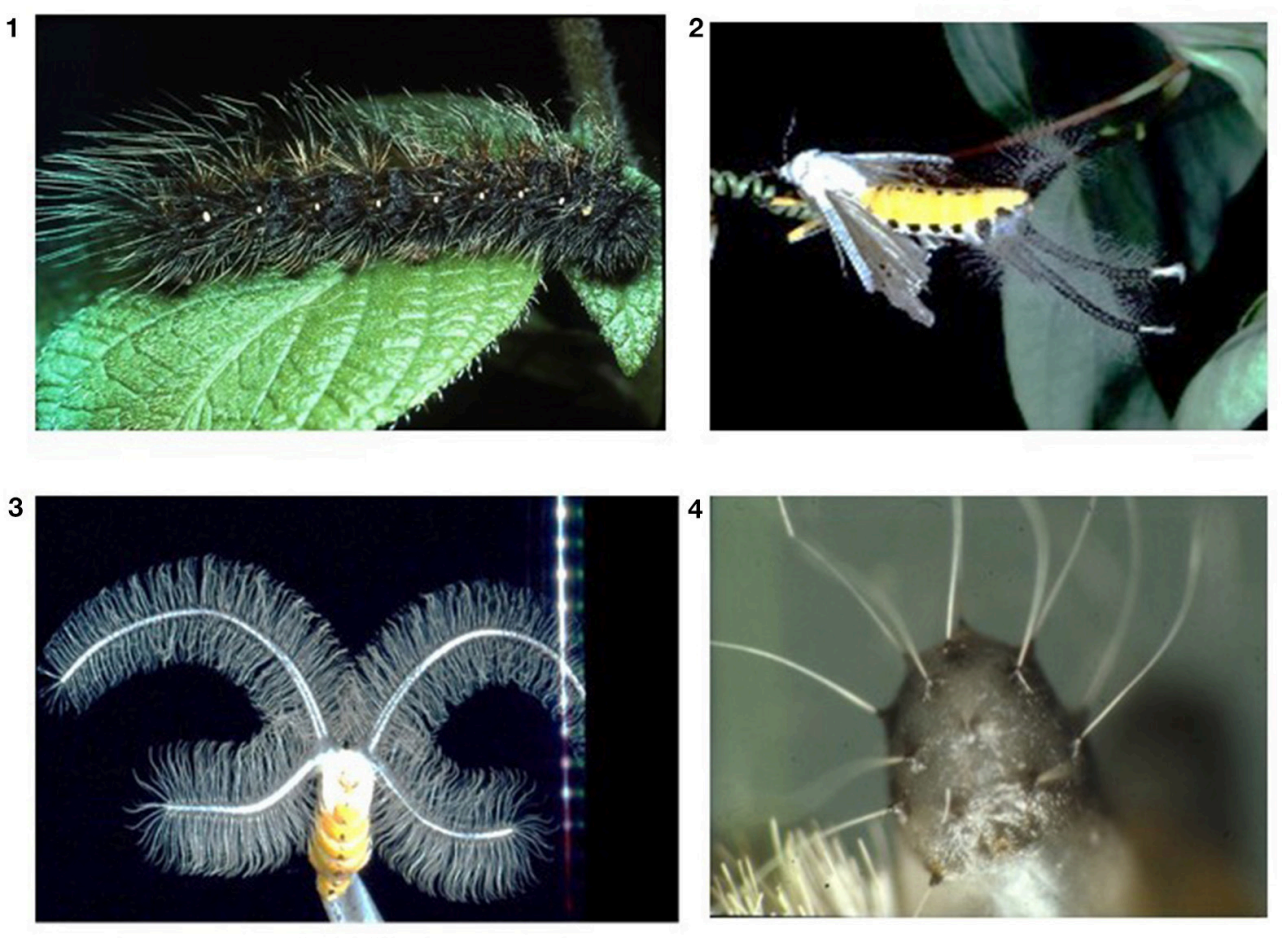
Influence of PAs on the development of male corema in *Creatonotos transiens*. (1) Caterpillar, (2) Adult male with inflated corema, (3) Large corema of a male whose larva had PA-rich food, (4) Minute corema of a male whose larva had no PAs in its food.

Males produce impressive coremata (these are inflatable sacks at the abdomen which are covered with many hairs) that are inflated during courtship and which will dissipate pheromones to attract female partners (Figure 1). Dietary PAs serve as a morphogen that induces the formation of coremata. If a caterpillar did not obtain PAs, then only very small coremata will develop in the imagines (Figure 1; Schneider et al., 1982; Boppré, 1986). Thus, the more PAs were ingested, the bigger the corema (von Nickisch-Rosenegk et al., 1990). It seems that co-evolution proceeded even a step further in this system (Schneider, 1992). The pheromones that are dissipated via the coremata, consist of hydroxydanadial (and others) that is derived from dietary PAs (Boppré, 1986; Wink et al., 1988; Schulz et al., 1993; Schulz, 1998). And evidence shows that female moths like males with an abundant PA perfume. And for good reason: we detected that the male spermatophore was also filled with dietary PAs that were transferred as a nuptial gift during copulation to the female increasing the PA contents of the eggs. Thus, males can contribute to the fitness of their offspring. Hydroxydanaidal that is produced by many PA plants is also a signal for other PA insects (Bogner and Boppré, 1989). However, arctiid caterpillars have taste receptor neurons which are dedicated to the perception of PAs and PA-N oxides (Bernays et al., 2002, 2003).

As shown in Table 1, many PSMs modulate the activity of neuroreceptors in vertebrates; what about insects? Insects have similar neurotransmitters, such as acetylcholine, GABA, glutamate, histamine, tyramine, dopamine, octopamine, and serotonin (Vleugels et al., 2015) indicating that neuroreception and corresponding mechanisms are evolutionary old features. Octopamine in insects is similar to noradrenaline in mammals (Vleugels et al., 2015). It is likely, that at least some of the PSMs listed in Table 1 as modulators of neuroreceptor activity for vertebrates, will also work on insect neuroreceptors. For example, there is evidence that insects lose their coordination when exposed to cocaine that binds to octopamine receptors. Serotonin receptors are expressed in the brain but also in the intestinal tract of animals. Serotonin is involved in the regulation of appetite, mood and emotion, sleep, sexual activity, pain, learning and memory (Vleugels et al., 2015). As serotonin agonist often induce euphoria and hallucinations in vertebrates, we can only speculate that maybe also insects react to serotonin receptor agonists (Vleugels et al., 2015). In vertebrates, PA bind to serotonin receptors (Schmeller et al., 1997). We do not know if this is also the case of serotonin receptors of insects that are also involved in the regulation of feeding, food choice and sleep (Vleugels et al., 2015). The addictive behavior of arctiid moths toward PAs, described above, would be plausible if this would be the case. This is an open question that needs to be addressed experimentally.

## Conclusions

Many PSMs interfere with neuroreceptors and neurotransmitters in vertebrates (Wink, 2000; Wink and Schimmer, 2010). Since neuroreception is on old evolutionary invention, insects share many neuroreceptors with vertebrates, but have tyramine and octopamine receptors in addition (Schneider, 1992; Vleugels et al., 2015). Many insects feed on a single or a few often phylogenetically related food plants. It has been demonstrated, that PSMs serve as olfactory cues for insects to identify their appropriate food plants (Brown and Trigo, 1995). The behavior of insects toward such chemical cues reminds of drug addiction in humans and other vertebrates. It is a challenge for physiologist to discover how PSMs modulate neuroreception, and thus food choice. Since many psychoactive PSMs affect the serotoninergic and dopaminergic system in vertebrates (Table 1), it would be worth studying their effects on insects and find out if they also trigger addiction and behavioral changes in invertebrates. There is good evidence for cocaine and nicotine that these alkaloids are active in this context (Barron et al., 2009; Baracchi et al., 2017).

## Author contributions

The author confirms being the sole contributor of this work and approved it for publication.

### Conflict of interest statement

The author declares that the research was conducted in the absence of any commercial or financial relationships that could be construed as a potential conflict of interest. The reviewer PFM and handling Editor declared their shared affiliation.

## Footnotes

^1^In this review, I often cite results from the research of myself or my co-workers. I am aware that many other scientists have also worked in this field and published thousands of scholarly papers, which could have been cited instead (apology to all colleagues, whom I did not cite). The review does not cover the complete literature that exists on this topic. If complete, the review would have been very long and outside the scope of the journal. Therefore, this invited article presents my personal and certainly limited view.
